# A random set scoring model for prioritization of disease candidate genes using protein complexes and data-mining of GeneRIF, OMIM and PubMed records

**DOI:** 10.1186/1471-2105-15-315

**Published:** 2014-09-24

**Authors:** Li Jiang, Stefan M Edwards, Bo Thomsen, Christopher T Workman, Bernt Guldbrandtsen, Peter Sørensen

**Affiliations:** Department of Molecular Biology and Genetics, Aarhus University, DK-8830 Tjele, Denmark; Center for Biological Sequence Analysis, Department of Systems Biology, Technical University of Denmark, DK-2800 Lyngby, Denmark

## Abstract

**Background:**

Prioritizing genetic variants is a challenge because disease susceptibility loci are often located in genes of unknown function or the relationship with the corresponding phenotype is unclear. A global data-mining exercise on the biomedical literature can establish the phenotypic profile of genes with respect to their connection to disease phenotypes. The importance of protein-protein interaction networks in the genetic heterogeneity of common diseases or complex traits is becoming increasingly recognized. Thus, the development of a network-based approach combined with phenotypic profiling would be useful for disease gene prioritization.

**Results:**

We developed a random-set scoring model and implemented it to quantify phenotype relevance in a network-based disease gene-prioritization approach. We validated our approach based on different gene phenotypic profiles, which were generated from PubMed abstracts, OMIM, and GeneRIF records. We also investigated the validity of several vocabulary filters and different likelihood thresholds for predicted protein-protein interactions in terms of their effect on the network-based gene-prioritization approach, which relies on text-mining of the phenotype data. Our method demonstrated good precision and sensitivity compared with those of two alternative complex-based prioritization approaches. We then conducted a global ranking of all human genes according to their relevance to a range of human diseases. The resulting accurate ranking of known causal genes supported the reliability of our approach. Moreover, these data suggest many promising novel candidate genes for human disorders that have a complex mode of inheritance.

**Conclusion:**

We have implemented and validated a network-based approach to prioritize genes for human diseases based on their phenotypic profile. We have devised a powerful and transparent tool to identify and rank candidate genes. Our global gene prioritization provides a unique resource for the biological interpretation of data from genome-wide association studies, and will help in the understanding of how the associated genetic variants influence disease or quantitative phenotypes.

**Electronic supplementary material:**

The online version of this article (doi:10.1186/1471-2105-15-315) contains supplementary material, which is available to authorized users.

## Background

Genome-wide association studies (GWAS) have been successful in the discovery of many novel genetic variants associated with human diseases. However, identifying the causative variant(s) is still a daunting task, as the mechanisms through which the variants influence disease or quantitative phenotypes are often unclear, particularly when the associated genes are of unknown function or have no clear connection to disease biology. Network-based approaches to prioritize candidate genes associated with human diseases have been proposed in a number of studies [1–6]. They build on the idea that mutations in the same gene or mutations in different members of a gene complex, which here are defined as a set of genes sharing (the deleted) functional identity, may lead to similar disease phenotypes. The functional identity of a gene complex can be co-expression patterns, or functional association of proteins in physical complexes or in pathways. This means that, once a gene complex with members involved in one disease has been identified, the other members of the complex become candidates for having a biological relationship with the same disease.

Thus, a method of disease gene prioritization is to search for genes or gene complexes that have a phenotypic profile similar to the phenotypic profile of the target disease. The disease phenotype can be described as the disease characteristics such as pathogenesis and clinical features. Biomedical records in the life sciences (e.g., OMIM, GeneRIF, PubMed) provide reviewed facts under a wide variety of biological conditions. A global examination of biological textual data will thus establish the phenotypic profile of genes with respect to their connection to disease biology. The phenotypic profile of a gene, referred to as the gene-associated phenotype, can be obtained by large-scale text-mining of biomedical records using information extraction and retrieval techniques, and filtering the biomedical terms with specific vocabularies such as that from the Unified Medical Language System (UMLS) [7].

A potential bottleneck in phenotypic profiling using text-mining of biomedical records is that currently the candidate genes can be of unknown function or have no clear connection to known disease biology. However, network-based inference may partially alleviate this problem, because the phenotypic profile of a candidate gene will be based on all phenotypes linked to the genes in a protein complex that consists of physical interactions or functional associations, rather than just to the gene itself.

This poses the need for a method that can summarize the relevance signals between the disease and candidate complex as a whole. Because the information to be integrated in this approach (i.e., protein complexes, biomedical text records, and associations between genes and textual records) is frequently updated and the choice is optional (e.g., the use of PubMed abstracts instead of OMIM records), it also requires a method that easily adapts to these factors. A promising method is based on a random-set scoring model used for gene-set enrichment analysis of genome-wide expression data [8]. It can be used to compute a score per gene or per complex representing an overall enrichment signal for the association of the candidate gene with the disease. It is computationally fast [9] and it can be calibrated in a number of ways (e.g., few gene-associated phenotypes that are highly similar to the disease phenotype or many gene-associated phenotypes moderately similar to the disease phenotype), providing a flexible and powerful way to quantify the relevance of the candidate gene to the disease.

The network-based phenotypic profile of a candidate gene depends on several factors. These include the source or the type of the biomedical records and the type of vocabularies [9, 10], such as Medical Subject Headings (MeSH) [11] and International Classification of Diseases (ICD), as well as the stringency of protein associations to define the protein complexes. These factors may affect disease gene prioritization regardless of the quantification method used. Examining the influences of these factors will provide a thorough evaluation of the prioritization performance of the random-set scoring model.

The objectives of this study were 1) to implement and validate a random-set scoring model to quantify the disease relevance of genes in a network-based disease gene-prioritization approach; 2) to investigate the influence on the model of protein association validity and the type of gene-associated phenotypic profile; and 3) to apply this model to identify and rank human genes on a genome-wide scale according to their phenotypic relevance to a wide range of human diseases.

## Results

We implemented and validated a random-set scoring model for a network-based gene prioritization approach. This approach uses biomedical records (e.g., OMIM, PubMed, and GeneRIF) as phenotypic profile for candidate genes to infer their association with diseases. The candidate gene is prioritized as a gene complex based on the physical and functional protein-protein interaction (PPI) network from STRING [12–18]. We validated our approach using the known disease and gene relationships in the OMIM database. Several of the key factors in the prioritization approach were investigated including the influence of the PPI confidence score threshold, distinct sources of gene-associated phenotypes and different vocabularies. We also compared the prioritization ability of the random-set scoring model with two alternative network-based prioritization approaches. Finally, the approach was used for a global ranking of all human genes according to their relevance for a range of human diseases in OMIM.

### Influence of protein-protein interaction (PPI) confidence scores

**Figure 1:**
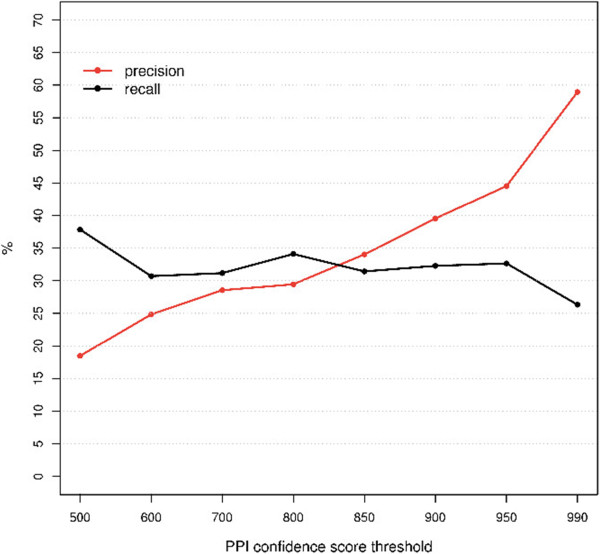
**Performance of the approach using different protein-protein interaction (PPI) confidence score thresholds.** The influence of different PPI thresholds on the precision (red) and recall (black) is shown. The precision (*y*-axis) and recall (*y*-axis) were determined for each PPI threshold (*x*-axis) at the maximal Matthews correlation coefficient (MCC).

**Figure 2:**
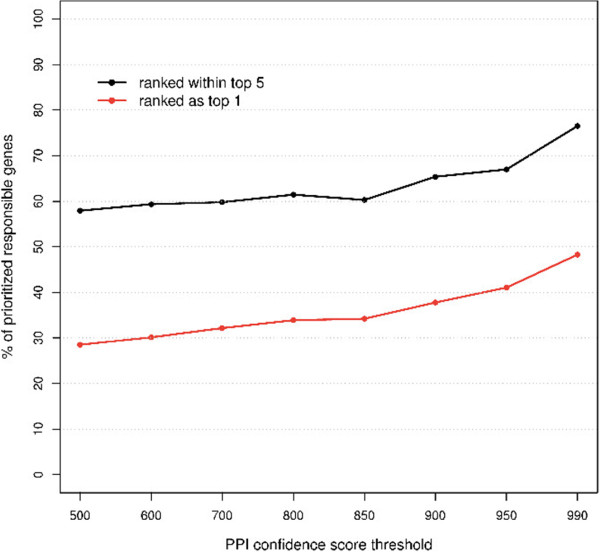
**Influence of protein-protein interaction (PPI) thresholds on the prioritization of causal genes in the test sets.** The proportion (*y*-axis) of prioritized test-sets where causal genes were ranked within the top five (black) or top one (red) is shown according to different PPI confidence score thresholds (*x*-axis).

We investigated the influence of protein complexes defined with different confidence thresholds on the performance of our network-based prioritization approach. The prioritization was examined with various confidence scores (500–990) of protein complexes, corresponding to different levels (median to high) of PPI quality (0.75–0.99) in STRING. The result (Figure 1) showed that, at the maximum Matthews correlation coefficient (MCC) (see Additional file 1), the precision of the method increased from 0.18 to 0.59, positively correlated with the PPI confidence scores. The sensitivity (recall) of the method was consistent, ranging between 0.31 and 0.34, using various protein complexes; only when the confidence threshold was relaxed to 500 or restricted to 990 did the sensitivity increase to 0.38 and decrease to 0.26, respectively. We then compared the number of highly ranked causal genes for different PPI confidence thresholds (Figure 2). The proportion of causal genes that could be ranked in the top one (top five) was between 0.28 (0.58) and 0.48 (0.77). Increasing the PPI confidence threshold led to a larger percentage of causal genes being highly ranked. However, with higher PPI confidence scores the proportion of causal genes that could be prioritized decreased from 0.89 to 0.45, primarily because of the availability of PPIs dependent on the confidence thresholds (see Additional file 1).

### Influence of gene-associated phenotype sources and phenotype vocabulary filters

**Figure 3:**
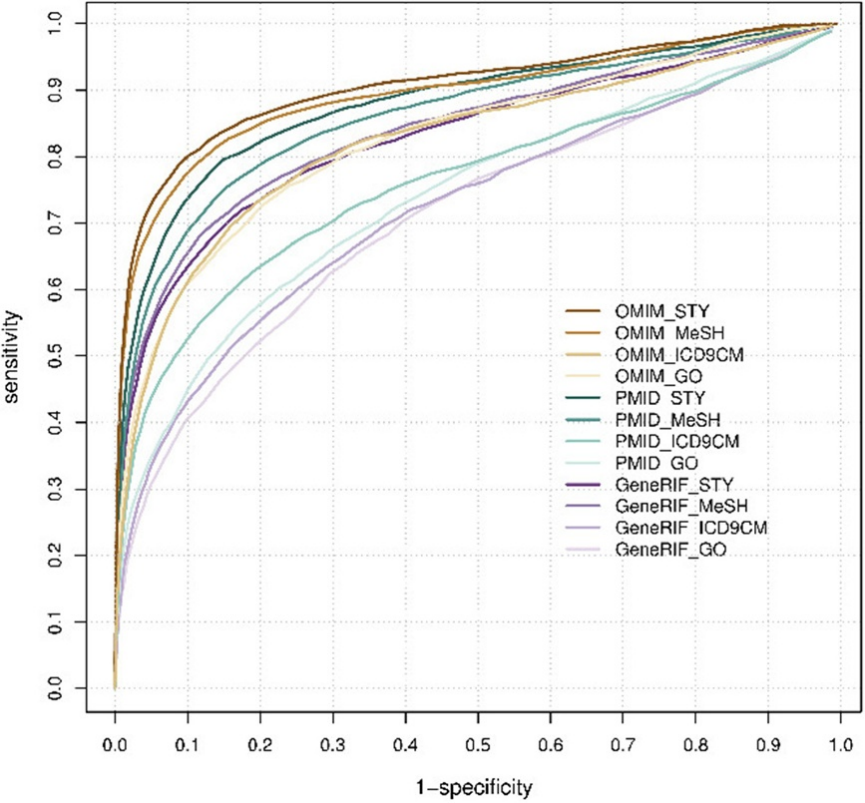
**Receiver operating characteristic (ROC) curves of prioritizations using different phenotype sources and vocabulary filters.** Each ROC curve represents the prioritization performance when combining a specific gene-associated phenotype with a vocabulary filter. The phenotype sources were OMIM (brown), PubMed (green), and GeneRIF (purple). The vocabulary filters were STY, MeSH, ICD9CM, and GO (colored from dark to light accordingly).

The biomedical records from three different databases (OMIM, PubMed, and GeneRIF) together with four vocabulary filters (MeSH, ICD ninth revision, clinical modification (ICD9CM), Gene Ontology (GO), and Semantic Type (STY) defined 12 sets of phenotypic profiles (see Additional file 1). Our overall evaluation showed that the random-set scoring model was an accurate and reliable predictor for disease gene prioritization. The area under the receiver operating characteristic (ROC) curve (AUC) was between 0.70 and 0.90 (Figure 3 and Table 1) for all the phenotypic profiles. Prioritization performance went in decreasing order for OMIM, PubMed, and GeneRIF. For the vocabulary filters, STY and MeSH were superior to ICD9CM and GO in revealing disease gene associations. The best performance, with an AUC of 0.85–0.90 (Table 1), was based on using OMIM records or PubMed abstracts with STY or MeSH.

**Table 1:** **Comparison of AUC (area under the curve), precision, and recall using different sources for the gene-associated phenotypes and phenotype vocabulary filters**

	OMIM			PubMed			GeneRIF		
	AUC	Precision	Recall	AUC	Precision	Recall	AUC	Precision	Recall
**STY**	0.90	0.52	0.48	0.87	0.40	0.43	0.82	0.40	0.32
**MeSH**	0.89	0.48	0.49	0.85	0.36	0.38	0.83	0.36	0.37
**ICD9CM**	0.81	0.31	0.38	0.75	0.31	0.28	0.71	0.35	0.19
**GO**	0.82	0.38	0.30	0.73	0.32	0.18	0.71	0.23	0.19

AUC, area under the curve.

The gene prioritizations were consistent across different phenotypic profiles. For example, our model ranked 50% (OMIM), 40% (PubMed), and 40% (GeneRIF) of the prioritized causal genes as the top candidates (Additional file 1: Table S10) according to the different phenotypic profiles (MeSH terms). Impressively, at least 57% of these cases were accurately predicted by all types of biomedical records. When taking only the causal genes that were ranked in the top five, the proportion was even higher (75%). When examining the prioritization results from any two phenotypic profiles, the common set of top-ranked causal genes accounted for a minimum of 39–82% between biomedical records, and 28–91% between vocabulary filters (Additional file 1: Tables S8 and S9). When applying protein complexes defined by different stringencies, the prioritization was generally more robust between two similar confidence scores than between two that were substantially different from each other (e.g., between 500 and 990) (Additional file 1: Figure S4).

### Comparison with other methods

We compared the prioritization ability of our random-set scoring model with that of two other network-based prioritization approaches that rely on gene-associated phenotypic profiles: a Bayesian prediction model proposed by Lage et al. [4] and a regression prediction model, CIPHER-DN, proposed by Wu et al. [6]. We estimated the recall and precision specifically for these comparisons using our matching test-sets. One group consisted of 84% (1177/1404) of the test cases from Lage et al. and the other consisted of 83% (1193/1444) of the test cases from Wu et al. The protein networks used in these approaches were restricted to protein interaction data that did not include the co-occurrence of genes in the biomedical literature. Our approach is based on protein complexes retrieved from the STRING database, which integrates both physical interactions and predicted protein associations based on multiple information sources, including text-mining of PubMed records for the co-occurrence of two genes. To assess the influence of gene co-occurrence in the literature, we recalculated the PPI confidence scores independent of the text-mining evidence (see Additional file 1). Table 2 shows the prioritization performance of the three different approaches. Our model demonstrated higher recall (0.43 versus 0.21) and higher precision (0.56 versus 0.45) than the Bayesian prediction approach. When adding the predicted interactions based on text-mining of the biomedical literature, the superiority of our method became more obvious. Our approach worked almost equally as well as the regression prediction approach (recall: 0.57 vs. 0.55; precision: 0.52 vs. 0.55) when the evidence from text-mining was included. When the protein complexes were defined excluding the text-mining evidence, we observed a decrease in recall whereas precision remained largely unaffected.

**Table 2:** **Comparison with other network-based approaches**

***Testing sets***	***Evaluation metrics***	***Random-set scoring model***		***Bayesian/regression method***
		*PPI evidence incl. co-mention	PPI evidence excl. co-mention	
Bayesian	Recall	0.55	0.43	0.21
	Precision	0.58	0.56	0.45
Regression	Recall	0.57	0.43	0.30 − 0.55
	Precision	0.52	0.47	0.73 − 0.55

*PPI, protein-protein interaction.

### Global ranking of human genes according to their disease relevance

We performed a genome-wide phenotypic screen (Additional file 1 in project website) on 19,032 human genes to predict their relevance to a range of disorders represented in the OMIM database (3053 phenotype descriptions). For many of these disorders, the molecular basis is known and does not represent a unique locus. We pinpointed the responsible causal gene for the corresponding disorder as the top candidate in 8% of known heterogeneous relationships. We observed that 66% of causal loci were ranked in the top 1% across the whole genome for causality with the associated disorder. Our genome-wide predictions provide a global ranking list, thus suggesting novel yet-to-be-validated candidate genes for a large number of human disorders.

We identified 680 (3.6%) genes that were ranked at least once as the top candidate gene for another range of (1921) OMIM phenotypes for which no susceptible loci have been identified. The gene most frequently (78 times) top-ranked (for 4% of the phenotypes) was catechol-O-methyltransferase (*COMT*).

To evaluate the ability of our gene-prioritization model to identify known disease genes as well as to identify unknown susceptibility factors, we selected two neurodegenerative disorders: amyotrophic lateral sclerosis (ALS; OMIM id: 105400) and Parkinson disease (PD; OMIM id: 168600). For both disorders, several causal genes have been identified although additional genetic risk factors remain to be found [19, 20]. The positions of recognized disease genes according to the prioritization model for each disease were identified. Thus, ten ALS-associated genes *SOD1 (position 9)*, *FUS (position 34)*, *ANG (position 19)*, *TARDBP (position 1)*, *ALS2 (position 4)*, *VAPB (position 29)*, *OPTN (position 190)*, *VCP (position 46)*, *MAPT (position 32)*, *C9ORF72 (position 126*) were prioritized among the top 1%, while an additional four genes (*SETX (position 816)*, *FIG 4 (position 1024)*, *DAO (position 1441)*, *SPG11 (position 1085*) were among the top 7.5%. One gene *SIGMAR1 position 2809*) occurred among the top 15%, while only one of the known ALS-associated genes, *UBQLN2 (position 16068)*, failed to be highly prioritized by our approach. For PD, the six top-ranked genes, *ATP13A2 (position 1)*, *LRRK2 (position 2)*, *PINK1 (position 3)*, *PARK2* also known as *PRKN (position 4*), *PARK7*also known as *DJ1 (position 5*), and *SNCA (position 6)* are all recognized disease genes, whereas additional genes linked to PD, *UCHL1* and *HTRA2*, were ranked in positions 10 and 138, respectively [21]. Among the highly prioritized genes in which mutations have not yet been identified, we observed a number of highly interesting genes, including *CCS* (position 2), *RNF19A* (position 5), *DERL1* (position 6), and *XRN2* (position 10) for ALS, and *KLK6* (position 7), *SLC6A3* (position 8), and *TPPP* (position 9) for PD. The possible pathogenic roles of these genes are dealt with in the Discussion.

## Discussion

We have implemented and validated a network-based disease gene-prioritization approach using a random-set scoring method. This approach prioritizes candidate disease genes using protein complexes and text-mining of the biomedical literature. We examined the influence of the key parameters of the approach, including the quality of the PPI information and the use of different sources of gene-associated phenotypes and vocabulary filters. Our implementation has a great transparency as it relies on publicly available data including PPI from the STRING database and biomedical publications from the National Center for Biotechnology Information (NCBI) databases. We used our approach to conduct a global ranking of all human genes for phenotypic association with a broad range of human diseases. These results provide a unique resource for the biological interpretation of results from GWAS, and will help understand how the associated genetic variants influence disease or quantitative phenotypes. Overall, we have shown that our network-based approach provides a powerful and flexible tool to identify and rank candidate disease genes.

### Assessment of our network-based gene-prioritization approach

Comparison of the random-set scoring method with the Bayesian prioritization model [4] and the regression prioritization model [6] sheds light on the overall performance of network-based gene-prioritization approaches. The PPI data used in our study was from STRING, which is a public and comprehensive database that integrates various molecular interactions from other repositories including MINT (Molecular INTeraction database) [22], HPRD (Human Protein Reference Database) [23], and BIND (Biomolecular Interaction Network Database) [24]. By applying the original STRING data, we ensured the transparency and reliability of our approach. The STRING data includes both physical interactions and functional or predicted interactions derived from, for example, text-mining of the biomedical literature. The usefulness of text-mining of the biomedical literature in STRING is to enable searching for the co-occurrence of two biological entities (e.g., genes or proteins) in a textual context, and thereby establish an association between them. In contrast, the text-mining of the biomedical literature used in this study for gene prioritization was to convert a text record into a list of disease-relevant biomedical terms that phenotypically characterize the linked gene. These are two different applications of text-mining techniques for biomedical text data. Our result indicates that the use of text-mining in identifying the co-occurrence of two genes in biomedical text could provide new knowledge on PPIs, which could improve disease gene identification.

Our scoring method is very similar to gene set enrichment analysis (GSEA) commonly used in expression analyses where gene-level scores (i.e. differential expression levels) are used for detecting the enrichment signal. In our procedure the gene-level scores are correlations measuring the similarity between text (an individual document or a set of documents) linked to the gene and text linked to the disease. A summary statistic is subsequently computed by averaging the correlations of the genes to the disease phenotype in the complex. The averaging is superior when the gene being tested contains many gene-associated phenotypes that weakly correlates with the target disease phenotype. This characteristic is in line with the hypothesis that complex diseases or traits are influenced by multiple genes and other environmental factors. Furthermore quite a number of studies comparing various GSEA approaches and gene set summary statistics show that the mean/sum statistics used in our procedure yield overall very good results [25]. We believe that contrary to other highly parametric Bayesian approaches, that our random-set scoring method is a simple, but robust approach for disease gene prioritization.

### Influence of gene-associated phenotypes

Our approach has the option of choosing different types of biomedical records for gene-associated phenotypes, including those from OMIM, PubMed, and GeneRIF. We showed that the source influenced the performance of the model. The use of OMIM records resulted in the highest recall and precision when tested on the human diseases present in the OMIM database. This was not surprising because, using OMIM, the phenotype for the genes and the disease comprised the same type of data; OMIM is the primary source of phenotypic descriptions for human diseases in similar network-based prediction approaches [4, 6]. However, these other studies did not examine the effect of using other types of biomedical literature. OMIM records are curated text that reviews the relevant references, of which the majority is indexed publications in the PubMed database. PubMed comprises nearly half a million gene-phenotype associations that involve more than 28,000 human genes. This suggests that PubMed articles and abstracts provide global phenotypic information for gene prioritization on a genome-wide scale. Our results also support the importance and validity of PubMed as the best source of gene-associated phenotypes apart from OMIM. A great advantage of PubMed (and GeneRIF) is that it is not restricted to human studies, but also includes other organisms; the phenotypic and genetic discoveries based on experimental models (in, for example, the mouse) can provide great insight into human diseases. PubMed may capture the phenotype-genotype relationships more comprehensively than OMIM does. This has important implications for disease gene prioritization in livestock species [26], for which only a limited number of phenotypic descriptions have accumulated in disease databases (e.g., OMIA), although many more gene-associated phenotypes may exist in literature databases such as PubMed or GeneRIF. The performance evaluation using the set of known causal genes from OMIM could be potentially overestimated even if the associations between a test gene and diseases (including test disease) were excluded from validation study. This is because the PubMed abstracts may contain the information that a test gene is involved in a test disease (e.g. the association between the gene and disease could be mentioned in abstracts linked to proteins that interact with the protein encoded by the test gene). In addition the prioritization could perform modest in discovering novel disease genes, because these genes may have limited information in terms of phenotypes and PPI. Some study suggested using an older version of phenotypic source (e.g., PubMed) and newly discovered causal genes, which would give less biased estimation of the method [27] Alternatively, one can probably adjust the semantic similarity between phenotypes according to the publication years to control the bias.

### Influence of the PPI interaction score

Protein complexes defined by high confidence PPI scores increased the precision of our network-based disease gene-prioritization approach. Thus, our results support the fact that the genes likely leading to similar disorders are strongly connected. Meanwhile, the recall was maintained for most PPI confidence score thresholds (600–950). However, further increasing the confidence threshold reduced the number of genes for which a candidate complex could be identified. The influence of protein confidence score thresholds was stronger on non-causal genes than on known causal genes in test. This is likely because known disease genes are usually better characterized and more frequently studied, and therefore have more known protein-protein interactions. Particularly, those disease genes which are also essential genes show a strong trend to encode hub proteins [28]. Despite the high stringency, we could have included more PPIs, particularly those non-physical functional associations predicted in STRING. In practice, this may help detect less-studied disease genes. It is important to note that our method is not limited to genes with known PPIs, although our validation was entirely dependent on the interaction partners of the causal genes. As long as the gene itself has one or more gene-associated phenotypes, we could quantify the association with any disease phenotype. This is a clear advantage compared with some existing network-based disease gene-prioritization approaches. In particular, as more information about gene function and PPI data become available, we expect that network-based approaches will become ever more accurate and sensitive in their ranking of candidate genes.

### Global ranking of genes for human diseases in OMIM

We used our approach to generate a global ranking of all human genes according to their relevance to a large set of human diseases. These results provide a unique resource for the biological interpretation of results from GWAS and will help understand how the associated genetic variants might influence disease or quantitative phenotypes. In addition, the global ranking profile (Additional file 1 in project website) may help to identify a set of genes encoding for the proteins in the protein complex that have co-susceptibility/resistibility to a common set of disorders, and similarly, disease clusters that share relevant/irrelevant genes.

For a more detailed assessment of the ability of the gene-prioritization model to identify disease genes accurately, as well as to identify unknown susceptibility factors, we selected two complex disorders for closer scrutiny: ALS and PD. ALS is a neurodegenerative disease, usually of adult onset, caused by the loss of motor neurons in the brain and spinal cord. ALS is clinically characterized by progressive muscular paralysis, leading to respiratory failure and death usually within five years of diagnosis. A subgroup of ALS patients develops frontotemporal dementia. ALS is predominantly a sporadic disease, while 10% of cases are familial. To date, 13 genes and two chromosomal loci have been linked to ALS, two genes with ALS-frontotemporal dementia, and one gene with ALS-Parkinsonism-dementia complex [19]. Ideally, a prioritization model should rank all known causal genes in the top positions, which is essentially what we observed. Inspection of the prioritized genes showed that ten genes associated with ALS occurred among the top 1%, four genes were among the top 7.5%, one was in the top 15%, and only one gene was poorly prioritized. Importantly, the model also prioritized several genes that have not yet been annotated as being associated with ALS. One such gene was *CCS*, which encodes a copper chaperone for Cu, Zn-superoxide dismutase 1 (SOD1). CCS is essential for SOD1 activity because it delivers the copper cofactor to the enzyme and promotes oxidation of an intra-subunit disulfide bond, which is important for the structural stability of SOD1. *SOD1* mutations are responsible for up to 20% of the hereditary forms of ALS, and a hallmark of both familial and sporadic cases of ALS is aggregates of misfolded SOD1 protein [29]. Ccs-null mice have markedly reduced Sod1 activity and, like Sod1-knockout mice, exhibit reduced fertility and an increased sensitivity to paraquat [30]. Furthermore, Ccs-deficient mice showed an increased loss of motor neurons after facial nerve axotomy, similar to Sod1 mutants [31]. Intriguingly, loss of Ccs did not affect the time of onset or progression of motor neuron disease in Sod1 mutants, whereas in contrast, over-expression of Ccs in a Sod1-mutant background strongly accelerated neurological disease. This occurred without the formation of insoluble Sod1 aggregates, but generated a markedly mitochondrial pathology, suggesting that Ccs may modulate disease progression by affecting the subcellular distribution of Sod1 between the cytosol and mitochondria [32, 33].

Another putative disease gene that might be linked to ALS is *XRN2*, which was ranked at position 10. The Xrn2 protein possesses 5′–3′ exoribonuclease activity and is involved in the termination of transcription. Behind the elongating RNA II polymerase, the nascent transcript forms RNA-DNA hybrid structures (R-loops), in particular in the G-rich pause sequences downstream of the poly(A) signal. The R-loops are subsequently resolved by the RNA/DNA helicase activity of senataxin, which is encoded by the ALS-related disease gene *SETX* [34]. This is a critical step because it allows the Xrn2 exoribonuclease to degrade the transcript from the free 5′ end generated by cleavage of the RNA at the poly(A) site, leading to transcriptional termination and the release of free RNA polymerase II [35]. The neurodegenerative mechanisms associated with mutations in *SETX*, and possibly also *XRN2*, are presently not understood. It is noteworthy, however, that Xrn2 has been shown to interact with TDP43, which is also involved in various aspects of RNA transcription and processing [36]. Mutations in the gene encoding TDP43, *TARDBP*, have been found in both familial and sporadic ALS [37]. Together, these observations support the notion that aberrant RNA processing plays a role in ALS pathogenesis [38].

Failure to fold newly synthesized proteins in the endoplasmic reticulum (ER) can result in the accumulation of misfolded proteins in the lumen, which activates ER stress signaling to protect the cell from the adverse effects of protein accumulation or aggregation [39]. For example, protein mutations may prevent their correct folding, which leads to retrotranslocation of the terminally misfolded proteins into the cytosol, where they are subjected to ubiquitin-dependent degradation in the proteasome, a process known as ER-associated degradation (ERAD). However, prolonged activation of ER stress may induce cell death by apoptosis, and the ER stress response has been shown to be involved in the pathogenesis of a number of different diseases, including SOD1-related ALS [40]. Interestingly, two of the genes that were prioritized by our pipeline, *DERL1* and *RNF19A*, are implicated in the ERAD response to SOD1 mutations. Thus, DERL1 or derlin 1 (position 6) is most likely a component in the channel responsible for the retrotranslocation of proteins across the ER membrane, and derlin 1 has been demonstrated to interact specifically with mutated SOD1, apparently leading to the inhibition of ERAD and the induction of apoptotic death in motor neurons [41]. Likewise, the *RNF19A* gene product dorfin (position 5) is also implicated in protein degradation by virtue of its ubiquitin-ligase activity. Dorfin selectively ubiquitinates and degrades mutated Sod1 in a dorfin-mediated ubiquitin-proteasome pathway, thereby protecting neuronal cells against the toxic effects of mutated Sod1 [42]. Furthermore, overexpression of dorfin in a mouse model of ALS reduced the number of Sod1 aggregates in the spinal cord, reduced motor neuron degeneration, and increased the life-span of the mutant mice [43]. Finally, dorfin colocalizes with valosin-containing protein (VCP) in Lewy body-like inclusions composed of ubiquitinylated protein aggregates in both ALS and PD. The two proteins interact and the ATPase activity of VCP contributes to the ubiquitin ligase activity of dorfin [44]. Notably, mutations in *VCP* are associated with ALS [45]. Taken together, the tight association of derlin 1 and dorfin with the ER stress response and degradation of mutant Sod1 makes them strong candidates for ALS susceptibility.

PD is characterized by slow movements, rigidity, impaired balance, and tremor at rest, and the pathological hallmarks include degeneration of dopaminergic neurons in the substantia nigra and accumulation of protein inclusions or Lewy bodies within nerve cells of the substantia nigra and several other brain regions [21]. In addition to correctly identifying known disease-linked genes, our computational approach prioritized three unrecognized genes among the top ten candidates. Among these was *KLK6*, also called neurosin, which encodes a serine protease primarily expressed in nervous tissue. Interestingly, neurosin is able to degrade alpha-synuclein, which is a major constituent of Lewy bodies in the brains of PD patients as well as one of the known disease genes. Thus, fragmentation of alpha-synuclein by neurosin inhibits the polymerization and aggregation of alpha-synuclein, suggesting that neurosin may play a role in pathogenesis [46]. Our model further suggested an involvement for *SLC6A3*, a dopamine transporter, an obvious candidate risk factor considering that PD is associated with the loss of dopaminergic neurons. Indeed, loss-of-function mutations in *SLC6A3* have recently been linked to a complex movement disorder involving infantile Parkinsonism and dystonia [47, 48]. Finally, the *TPPP* gene, encoding the tubulin polymerization-promoting protein or p25alpha, was top-ranked on the candidate gene list for PD. The *TPPP* gene is specifically expressed in the brain; its exact biological function is unknown but it seems to modulate the organization and dynamics of the microtubular network by interacting with tubulin [49]. Interestingly, p25alpha is primarily expressed in oligodendrocytes; however, abnormal expression has been observed in affected nerve cells in PD and Lewy body dementia. Furthermore, p25alpha promotes the aggregation of alpha-synuclein and co-localizes with alpha-synuclein in neuronal Lewy body inclusions. This indicates that dysregulated expression of *TPPP* may contribute to an increased risk of PD [50].

## Conclusions

We have implemented and validated a network-based approach to prioritize genes for human diseases based on their phenotypic profile. We have devised a powerful and transparent tool to identify and rank candidate genes. Our global gene prioritization provides a unique resource for the biological interpretation of data from genome-wide association studies, and will help in the understanding of how the associated genetic variants influence disease or quantitative phenotypes.

## Methods

### Network-based gene-prioritization approach

The prioritization approach works as follows. For each candidate gene, a candidate complex is determined from PPI data in STRING. Each gene in the complex is then linked to its biomedical text records in the OMIM, PubMed, or GeneRIF databases. Text-mining is used to convert the biomedical text into a vocabulary list based on the UMLS. For each gene, the result is a vector of weighted counts of occurrence of each of the UMLS terms present in the biomedical text record. This vector defines a standardized gene-associated phenotype. Vectors for standardized disease phenotypes are determined in a similar way. In both cases, the terms used can be limited to specific vocabularies by applying different vocabulary control filters (e.g., MeSH, GO). A disease relevance score for each candidate gene is computed from the pair-wise semantic similarities between the disease phenotype and each of the gene-associated phenotypes in the complex. A random-set scoring model is used to calculate the disease relevance score. The random-set scoring model gives a z-score per gene complex that represents an overall enrichment signal for the association of the candidate gene with the disease. The z-score is used to determine whether the gene is associated with the disease, and is used to rank/compare genes for their disease relevance.

### Website

More information regarding this network-based approach can be found on the project website at https://djfextranet.agrsci.dk/sites/txtphenome/public/Pages/front.aspx. This site contains the R-package txtPhenome and data packages containing processed data from OMIM, GeneRIF, and PubMed, and genome-wide ranking of genes for relevance to OMIM disorders (Additional file 1). txtPhenome contains functions for dealing with the data in the data packages.

### Disease and gene-associated phenotypes

Disease and gene-associated phenotypes were obtained from text-mining of the biomedical text data (OMIM, GeneRIF, PubMed records). The phenotypic profile of the disease, referred to as the disease phenotype, was obtained from OMIM records, which include a textual description of the disease characteristics such as pathogenesis and clinical features. The biomedical text records linked to human Entrez gene identifiers were from three repositories: 1) the text field of OMIM records; 2) the titles and abstracts of PubMed articles; and 3) the text field of GeneRIF records. The links between the specific Entrez gene identifiers and the biomedical text records were obtained from the NCBI (ftp://ftp.ncbi.nih.gov/gene/DATA/). Additional file 1: Table S11 shows an overview of the biomedical records and the links to the genes in different repositories.

### Text-mining of biomedical records

The text field of the biomedical records from OMIM, PubMed, or GeneRIF was processed through the text-mining program MMTx (version V2.4.C) [51]. Each document was mapped into to a set of UMLS (release version: 2009AA) concepts/terms. UMLS is a metathesaurus comprising various vocabularies (58 sources in version 2009AA) including MeSH, ICD9CM, and GO. In addition, the UMLS defines 135 semantic types, which provide a consistent categorization of all concepts represented in the UMLS metathesaurus. The set of UMLS concepts obtained from each document was filtered in one of two ways: (1) by a specific vocabulary; or (2) by a group of semantic types. In (1), three UMLS vocabularies (MeSH, ICD9CM, and GO) were applied independently to the concept vector for each document. In (2), the concepts that belonged to a group of semantic types (see Additional file 1) were retained in the phenotypic vectors. The removed concepts were those that belong to semantic types (e.g., “governmental or regulatory activity”) that were clearly not relevant to biological phenotype. The concept volumes of different vocabulary filters are given in Additional file 1: Table S13. The frequencies of concepts within the document and the occurrences of concepts across documents were calculated. Finally the term frequency-inverse document frequency [52] was used to weight all the terms in each document.

The semantic similarity used in this study was the correlation between two documents. One was the disease-associated phenotype (an OMIM record) and the other was the gene-associated phenotype (an OMIM record, a PubMed abstract, or a GeneRIF record). The semantic similarity was computed as the cosine coefficient between the weighted term occurrences in a disease-associated phenotype and a gene-associated phenotype according to the Vector Space Model [53, 54]. The biomedical records from the three gene-associated phenotype sources and the four vocabulary filters defined 12 sets of phenotypic profiles. The number of associations between human genes and biomedical records was highest for the PubMed database with ~473,000 PubMed abstracts linked to more than 28,000 genes (Additional file 1: Table S11). The vocabulary volume within each of the vocabulary filters varied from tens of thousands (for GO or ICD9CM) to hundreds of thousands (for MeSH), to nearly one and a half million (for STY) (Additional file 1: Table S13). Because of the length of the records and vocabulary volumes, the concept size derived from the phenotypes varied considerably (Additional file 1: Table S12).

### Candidate complexes

The candidate complex for each gene was determined from PPI data obtained from the STRING database (version 8.1). STRING is a database and web resource dedicated to PPIs, which contains both physical and functional interactions [12, 15]. The Ensembl protein encoded by the candidate gene was used to retrieve first-order protein interaction partners in the network that associate directly with that protein. Each pair of protein associations from STRING was annotated with an interaction confidence score ranging from 150 (low credibility) to 999 (high credibility). Additional file 1 shows the counts of PPI pairs ordered by their credibility scores. We examined the random-set scoring model with candidate complexes defined by different confidence score thresholds (500–990), corresponding to various levels of association likelihoods (0.75–0.99).

### Disease relevance score of a candidate gene complex

To prioritize the disease candidate genes, a disease relevance score that quantifies the association strength between a candidate gene complex and the disease was proposed. This score was based on a random-set scoring model [8], which was originally applied to study gene set enrichment analysis. Analog to the expression profile, in the context of this study, the semantic similarities (the cosine coefficient) between the documents are regarded as the phenotype profile. The random-set scoring model measures the enrichment signal of the set of semantic similarities between the disease phenotype (narrative description) and the set of gene-associated phenotypes (narrative descriptions) linked to the candidate gene complex. The semantic similarity between the disease phenotype and a gene-associated phenotype is defined as *S*_*e*_. The overall association between a disease and a candidate gene complex is represented by a set of semantic similarities that contains *m* elements, where *m* is the number of biomedical records linked to the genes in the candidate complex. The unstandardized enrichment signal is defined as the mean of the semantic similarities in set *C* [8] , where *C* is the set of *m* biomedical records that linked to a specific gene candidate complex, and is a random set of *m* documents drawn from the entire cohort of *E* documents. Under the random-set scoring model [8], and thus conditional on element-level scores {*S*_*e*_} of semantic similarities between disease phenotype and all documents:
1

and
2

A standardized enrichment score [9] is then calculated as , representing the disease relevance score. Under the null hypothesis that says “no association between the gene-associated phenotypes and the disease phenotype”, the disease relevance scores have a normal distribution with zero mean and unit variance [8]. A large positive disease relevance score (z-score) favors the positive enrichment hypothesis: that the candidate gene (or candidate complex) is strongly associated with the disease.

### Validation of the approach

We validated our network-based gene-prioritization approach using the known disease and gene relationships in the OMIM database. We used 3395 test cases, each corresponding to a known relationship between a disease phenotype and a disease-“causing” gene in the OMIM database. The test cases were identified from the morbid map downloaded from the NCBI (ftp://ftp.ncbi.nih.gov/gene/DATA/). Each relationship between an OMIM descriptive entry and a human Entrez gene labeled with “phenotype” was referred to as a test case in which the OMIM record represented the disease phenotype and the linked Entrez gene was the true “causal” gene. The 3395 test cases represented 2525 known human disorders and 2135 unique human genes. The intention was to mimic a situation in which we have identified a genomic region where one or several genetic variants are known to be associated with a specific disease phenotype. The challenge was to identify which of the genes located in that genomic region was the most likely candidate gene harboring a causal mutation. For each test case, a set of candidate genes, referred to as the test set, was identified by choosing 50 genes upstream and 50 genes downstream of the true “causal” gene. If the true “causal” gene was located close to the telomere then the test set would include more genes in the opposite direction to ensure a set of 100 candidate genes for each test case.

Although the genomic regions were relatively large compared with the resolution power of GWAS, they allowed us to compare the performance of our approach directly with that of two alternative complex-based prioritization approaches: the Bayesian prioritization model [4] and the regression prioritization model [6]. Both of these approaches have been previously validated on the OMIM data. To ensure fair comparisons, we applied the same vocabulary filters (either STY or MeSH) and used the same test sets as in the original studies. We estimated the recall and precision of our approach using two groups of test sets. One group consisted of 1177 test cases used for the Bayesian approach and the other group consisted of 1193 test cases used for the regression approach. The protein complexes were defined with credibility scores above 900. The protein complexes used in the alternative approaches were restricted to PPI data that did not include the co-occurrence of genes in the biomedical literature. Our approach was based on protein complexes retrieved from the STRING database, which integrates both physical interactions and predictive protein associations based on different information sources, including text-mining of PubMed records for the co-occurrence of two genes. To assess the influence of the text-mining evidence on the prioritizations, we recalculated the confidence scores leaving out this specific channel of PPI data (see Additional file 1).

We investigated the influence of the PPI confidence score threshold using GeneRIF as the gene-associated phenotype and STY as the vocabulary control. We assessed the influence of different sources of gene-associated phenotypes and phenotype vocabulary filters using a minimal PPI confidence score of 900.

We measured the performance of our approach in two ways: 1) for each test set, we ranked the candidate genes according to the standardized z-score. We determined the rank of the true causal gene and counted the number of times that the true “causal” gene ranked at the top or was among the five highest-ranking genes across all test sets; 2) we selected z-scores derived from the null hypothesis distribution (see Additional file 1) at cumulative quantiles (from 0.1% to 100%) as discriminators (cut-offs) to classify whether a gene from a test-set was associated with the disease phenotype. True positives (tp) and false negatives (fn) were identified when the z-score of a true causal gene from a test-set was above or below the discriminator, respectively. True negatives (tn) and false positives (fp) were identified when the z-score of a candidate gene was below or above the discriminator, respectively. The prioritization performance of the approach was examined by recall: ; precision: ; MCC: ; the ROC curve (sensitivity vs. 1–specificity) [55] and the AUC.

### Application of our approach

We used our approach to conduct a global ranking of all human genes for their relevance to human diseases represented in the OMIM database. The complete set of human genes was retrieved using an R package (org.Hs.eg.db) that is based on the Entrez gene database. The protein complexes for human genes were collected from the STRING database, and the threshold for PPIs was restricted to 0.95 or higher, which corresponds to a confidence score of 900. The phenotypic profile of each gene complex was generated from PubMed articles (titles and abstracts). The remaining phenotypic concepts were specifically limited to MeSH terms. According to the approach described above, we ranked all the human genes and scored them for their relevance to a large number of disease phenotypes from the OMIM database, which comprised either a descriptive entry, usually of a phenotype, and did not represent a unique locus (3053 OMIM identifiers marked by “#”), or a description of a phenotype for which the Mendelian basis, although suspected, has not been clearly established or the separateness of this phenotype from that in another entry is unclear (1921 OMIM identifiers without a label).

## Electronic supplementary material

Additional file 1: **Methods and results in details.** (PDF 766 KB)
