# Supplementary material for: A random set scoring model for prioritization of disease candidate genes using protein complexes and data-mining of GeneRIF, OMIM and PubMed records
Source: BMC Bioinformatics. 2014 Sep 24;15(1):315. doi: 10.1186/1471-2105-15-315 (PMC4181406; doi:10.1186/1471-2105-15-315)
Supplement: Supplementary file 1 — Additional file 1: Methods and results in details. (PDF 766 KB) [file 12859_2013_6589_MOESM1_ESM.pdf]

Supplementary file

We implemented a random-set scoring model for complex-based gene-prioritization relying on phenotypic profile, and validated it using known gene-phenotype relationships from OMIM. For each entry, the textual description of the OMIM record was regarded as the phenotype of a disease, and the linked gene was the corresponding "causal" gene. We fitted a random-set scoring model to the semantic similarities between a candidate gene (based on its protein complex) and a disease (an OMIM record) to quantify the phenotypic relevance according to the phenotypic profile. The disease relevance was computed as a z-score, which represents an overall enrichment signal of the candidate complex for the relationship with the disease. The z-score can be applied directly to rank genes for their disease associations. It can also be used to predict whether a gene/gene complex is associated with a disease based on a null hypothesis distribution where morbidity between diseases and genes is not expected. Gradient cut-offs from the null hypothesis distribution were used to evaluate the prioritization performance.

Null hypothesis data

The null hypothesis distribution of z-scores was based on the phenotypic profiles of diseases and genes that are not associated. To generate the null hypothesis distribution, genes that were identified as not related to the 3395 tested phenotypes were selected in three steps: (1) sample a random chromosome from the genome after excluding the chromosome harboring the true responsible gene; (2) sample a random locus from the selected chromosome; and (3) centered on this locus, choose 100 genes in this genomic region. This resulted in 335,740 pairs of OMIM phenotypes and Entrez genes with no previously identified biological connection.

Matthews correlation coefficient (MCC)

At selected quantiles in the gradient, we used the corresponding z-scores from the null hypothesis distribution as cut-offs to determinate the true positives, false positive, true negatives, and false negatives, and eventually the MCC and receiver operating characteristic (ROC) curves. As shown in Figures S1 and S2, the MCC reached a maximum when the quantile of cut-off approached 100% (corresponding to a large z-score). The cut-off of z-score and quantile giving the maximum MCC with respect to various confidence score thresholds, sources of biomedical records, and vocabulary filters are shown in Tables S1 and S2.

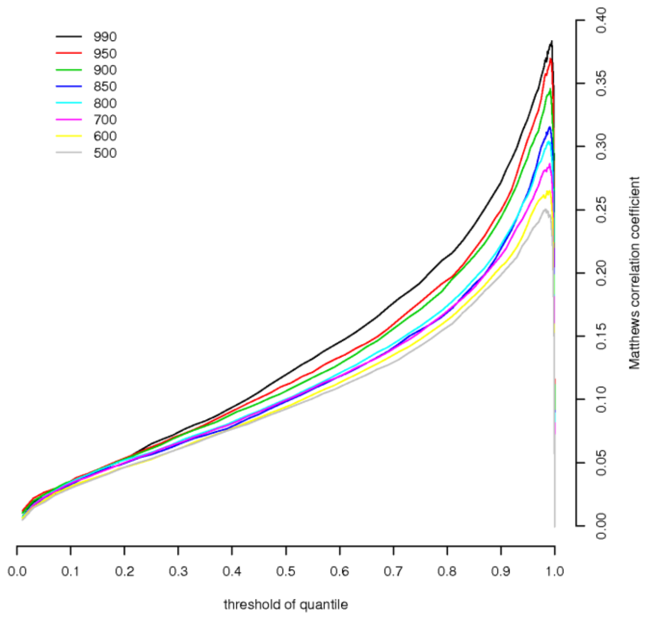

Figure S1 Matthews correlation coefficients at different cut-offs under each confidence score threshold.

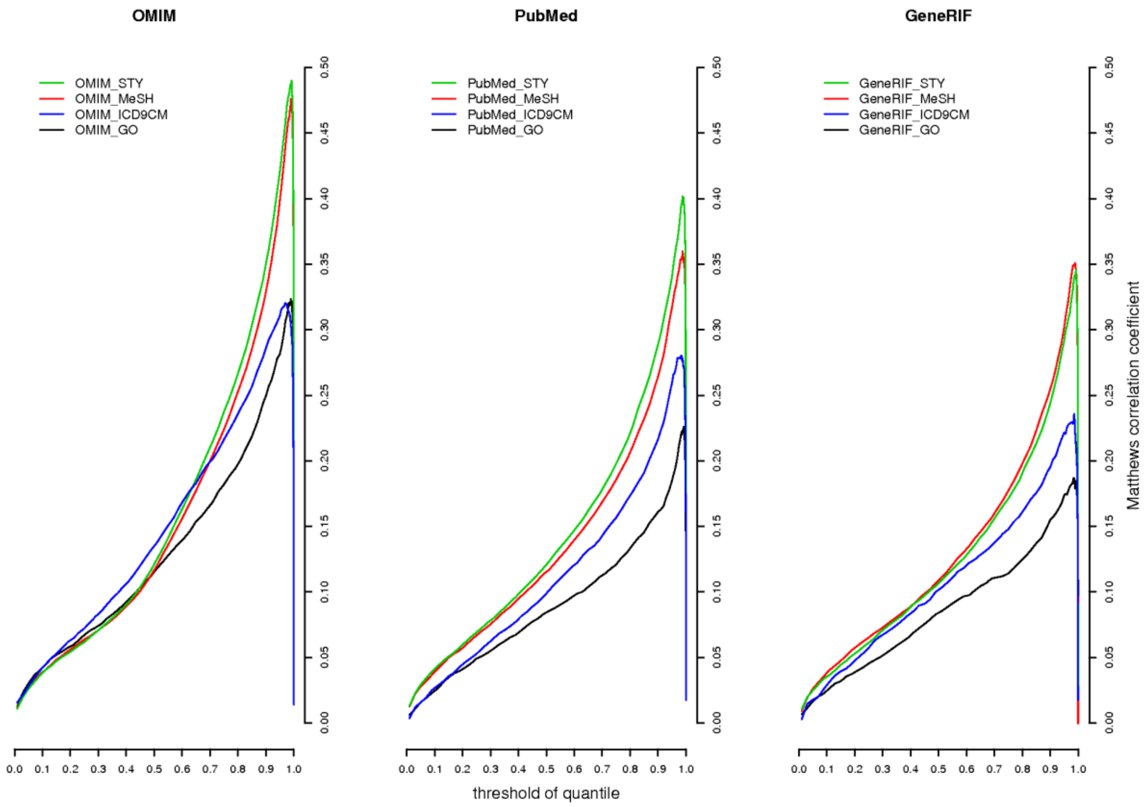

Figure S2 Matthews correlation coefficients at different cut-offs when using different phenotype sources and vocabulary filters.

Table S1 Matthews correlation coefficient (MCC) according to different confidence score thresholds for protein associations (phenotypic profile defined by GeneRIF and STY).

	Maximum MCC	Cut-off value (z-score) for max. MCC	Cut-off quantile for max. MCC
500	0.250	11.419	0.983
600	0.265	13.903	0.991
700	0.286	13.642	0.991
800	0.304	11.958	0.989
850	0.315	12.550	0.991
900	0.346	12.297	0.992
950	0.369	11.524	0.992
990	0.383	11.484	0.995

Table S2 Overview of Matthews correlation coefficient (MCC) for different phenotype sources and vocabulary filters (protein complexes defined by a confidence score of 900).

	Maximum MCC	Cut-off value (z-score) for max. MCC	Cut-off quantile for max. MCC
OMIM_STY	0.490	6.233	0.992
OMIM_MeSH	0.476	5.582	0.990
OMIM_ICD9CM	0.320	3.339	0.970
OMIM_GO	0.323	4.443	0.989
PubMed_STY	0.402	26.884	0.988
PubMed_MeSH	0.360	23.560	0.988
PubMed_ICD9CM	0.280	5.977	0.984
PubMed_GO	0.226	15.282	0.992
GeneRIF_STY	0.346	12.297	0.992
GeneRIF_MeSH	0.351	9.315	0.988
GeneRIF _ICD9CM	0.236	4.277	0.986
GeneRIF_GO	0.187	5.179	0.985

Protein complexes from STRING

The confidence score for human protein-protein interactions (PPIs) in STRING version 8.1 ranges from 150 to 999 (Figure S3). In our study, we applied a set of scores (500–990), corresponding to the approximate probabilities (0.75–0.99) of the two proteins being associated, to define the protein complex for every candidate gene. Each complex could consist of several proteins (high confidence) to tens of proteins (low confidence) depending on the stringency applied (Table S3). Not all the genes have a corresponding protein complex

at a given specific PPI threshold. In general, the higher the confidence score, the more candidate genes were obscured (Table S3). Correspondingly, the average number of phenotypes (for example, GeneRIF records) that were linked to a candidate complex reduced from 1894 to 342, because the size of the complex became smaller when using a more stringent criterion. The susceptibility to the loss of protein complexes and phenotypes was more evident for the rest of the candidate genes compared with for the true responsible genes in the same testing sets.

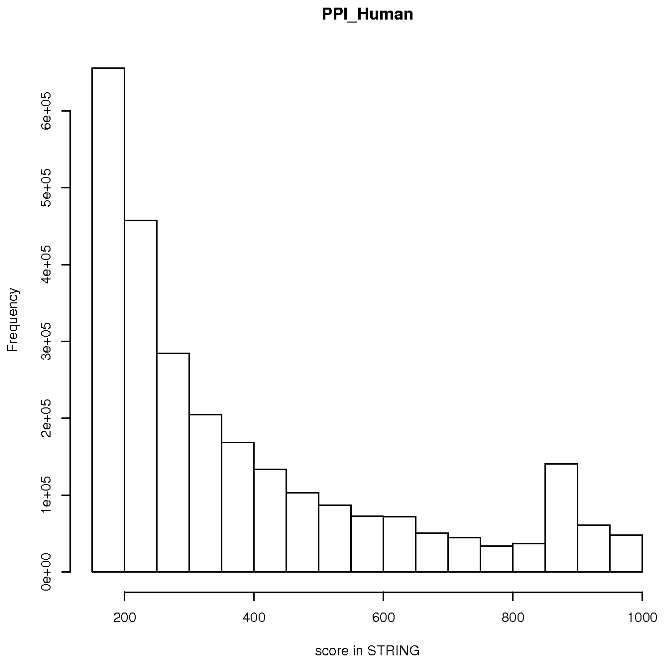

Figure S3 Frequency of human protein-protein interactions according to different confidence scores in STRING v_8.1.

Table S3 Candidate complexes defined with different confidence score thresholds for protein-protein interactions (PPI) in STRING.

PPI score threshold	*avg. size of complex*	*% of candidate genes associated with other proteins*
**500**	40	67%
**600**	31	64%
**700**	27	56%
**800**	24	49%
**850**	22	46%
**900**	12	40%
**950**	7	30%
**990**	4	18%

For all types of associations (both physical and predicted PPIs) in STRING, after assignment of the association scores and transfer between species, a final ‘combined score’ between any pair of proteins was computed. To assess the effect on the prioritization approach of protein networks that were derived from text-mining evidence in STRING, we examined our model using two sets of protein complexes: (1) the original data from STRING that were based on all PPI channels; and (2) data for which the text-mining evidence was omitted. The latter was computed in the same way as the original STRING data, under the assumption of independence for the various sources, in a naïve Bayesian fashion. To each channel, a 'prior' was added to account for the probability that two randomly picked proteins were interacting. Before combing the channels, the 'prior' was removed and was added back to the combined score, as described below:

1. For each of the scores for the individual channels (s_i), remove the prior (p = 0.063)

$$s_i^{no\ prior} = \frac{s_i - p}{1-p}$$

2. Combine the scores of the channels

$$s_{total}^{no\ prior} = 1 - \prod_i \left(1 - s_i^{no\ prior}\right)$$

3. Add the prior back (once)

$$s_{total} = s_{total}^{no\ prior} + p \cdot \left(1 - s_{total}^{no\ prior}\right)$$

We examined the prioritization ability of the model based on these complexes with various confidences. The credibility of the protein complexes was positively correlated (Pearson correlation: 0.97) with the accuracy (MCC) of our prioritization model. Moreover, the AUC of the prediction remained intact at 0.80–0.83 (Table S4).

Table S4 Comparison of areas under the curve using different protein-protein interaction score thresholds.

500	600	700	800	850	900	950	990
0.83	0.83	0.82	0.82	0.81	0.82	0.81	0.80

Prioritization is dependent on data availability

If a gene was not prioritized, this was mainly because (1) no protein complex could be identified from the PPIs retrieved at a given confidence score threshold; (2) there was no biomedical record linked to any member of the protein complex; or (3) similarity between the disease- and gene-associated phenotypes could not be determined because of term deprivation resulting from vocabulary control.

The effect of the protein confidence score threshold was evident. It led to the causal genes in only 45% of the testing sets being prioritized when the complexes were constrained by a confidence score above 990. In contrast, the proportion of prioritized causal genes increased to 89% if the threshold was dropped to 500. Increasing the threshold for the protein complexes led to fewer causal genes being prioritized. Table S5 shows the influences on both causal and co-located candidate genes according to different confidence scores.

A lack of links to biomedical records caused minor losses. On average, less than 1% of testing sets were affected by this, and this was exclusively because no phenotypes were available for the causal complexes. Vocabulary controls affected the data to various degrees; this appeared to be a consequence of interaction between different phenotype sources (Table S6). In general, STY and MeSH barely limited the prioritization of causal genes. In contrast, ICD9CM and GO had more effect on failing the causal genes in the testing sets (Table S6).

The influence of protein confidence score thresholds was stronger on candidate genes than on known causal genes (Table S5). The effect of phenotype source and vocabulary control was similar between the two (Table S6).

With the amount of publicly available data increasing at a considerable rate, it will become increasingly feasible to identify many validated PPIs and links between genes and phenotypes from biomedical publications. Thus, the failure of prioritization due to data availability will be overcome. Vocabulary control customizes the scope of the phenotypic profile of genes for disease relevancy. Thus, the decrease in the number of prioritized causal genes due to vocabulary filtering is not a failure directly linked to the prioritization method itself. In this study, we evaluated the performance of our prioritization approach based on the testing sets in which causal genes could be prioritized.

Table S5 Proportion of genes that could be prioritized: the influence of confidence score thresholds.

	Causal genes			Candidate genes		
	PPI	GeneRIF	STY	PPI	GeneRIF	STY
500	0.8934	0.8928	0.8919	0.6757	0.6676	0.6631
600	0.8895	0.8878	0.8863	0.6504	0.6398	0.6339
700	0.8742	0.8695	0.8675	0.5650	0.5453	0.5388
800	0.8533	0.8474	0.8439	0.5002	0.4913	0.4865
850	0.8365	0.8309	0.8274	0.4703	0.4629	0.4604
900	0.7965	0.7900	0.7838	0.4059	0.3983	0.3919
950	0.6972	0.6875	0.6810	0.3138	0.3083	0.3044
990	0.4545	0.4518	0.4462	0.1705	0.1664	0.1637

PPI, protein-protein interaction

Table S6 Proportion of genes that could be prioritized: the influence of phenotype sources and vocabulary filters.

	Causal genes			Candidate genes		
	PPI 900	Phenotype	Vocabulary	PPI 900	Phenotype	Vocabulary
OMIM_STY	0.7965	0.7956	0.7953	0.4059	0.4019	0.4006
OMIM_MeSH	0.7965	0.7956	0.7950	0.4059	0.4019	0.3999
OMIM_ICD9CM	0.7965	0.7956	0.6786	0.4059	0.4019	0.2547
OMIM_GO	0.7965	0.7956	0.6934	0.4059	0.4019	0.3159
PubMed_STY	0.7965	0.7965	0.7962	0.4059	0.4059	0.4052
PubMed_MeSH	0.7965	0.7965	0.7962	0.4059	0.4059	0.4052
PubMed_ICD9CM	0.7965	0.7965	0.7001	0.4059	0.4059	0.3102
PubMed_GO	0.7965	0.7965	0.7193	0.4059	0.4059	0.3498
GeneRIF_STY	0.7965	0.7900	0.7838	0.4059	0.3983	0.3919
GeneRIF_MeSH	0.7965	0.7900	0.7791	0.4059	0.3983	0.3865
GeneRIF _ICD9CM	0.7965	0.7900	0.5246	0.4059	0.3983	0.1899
GeneRIF_GO	0.7965	0.7900	0.5968	0.4059	0.3983	0.2631

PPI, protein-protein interaction

Semantic types used as the STY control

In total, 107 out of 135 semantic types from the Unified Medical Language System (UMLS) were selected as vocabulary filters for STY (Table S7).

Table S7 Semantic types selected as STY filters.

No.	TUI	STY	No.	TUI	STY
1	T020	Acquired Abnormality	55	T058	Health Care Activity
2	T052	Activity	56	T125	Hormone
3	T100	Age Group	57	T016	Human
4	T003	Alga	58	T068	Human-caused Phenomenon or Process
5	T116	Amino Acid, Peptide, or Protein	59	T129	Immunologic Factor
6	T087	Amino Acid Sequence	60	T130	Indicator, Reagent, or Diagnostic Aid
7	T011	Amphibian	61	T055	Individual Behavior
8	T190	Anatomical Abnormality	62	T037	Injury or Poisoning
9	T017	Anatomical Structure	63	T197	Inorganic Chemical
10	T008	Animal	64	T009	Invertebrate
11	T195	Antibiotic	65	T034	Laboratory or Test Result
12	T194	Archaeon	66	T059	Laboratory Procedure
13	T007	Bacterium	67	T171	Language
14	T053	Behavior	68	T119	Lipid
15	T123	Biologically Active Substance	69	T015	Mammal
16	T038	Biologic Function	70	T074	Medical Device
17	T091	Biomedical Occupation or Discipline	71	T048	Mental or Behavioral Dysfunction
18	T122	Biomedical or Dental Material	72	T041	Mental Process
19	T012	Bird	73	T063	Molecular Biology Research Technique
20	T029	Body Location or Region	74	T044	Molecular Function
21	T023	Body Part, Organ, or Organ Component	75	T085	Molecular Sequence
22	T030	Body Space or Junction	76	T070	Natural Phenomenon or Process
23	T031	Body Substance	77	T191	Neoplastic Process
24	T022	Body System	78	T124	Neuroreactive Substance or Biogenic Amine
25	T118	Carbohydrate	79	T114	Nucleic Acid, Nucleoside, or Nucleotide
26	T088	Carbohydrate Sequence	80	T086	Nucleotide Sequence
27	T025	Cell	81	T057	Occupational Activity
28	T026	Cell Component	82	T090	Occupation or Discipline
29	T043	Cell Function	83	T109	Organic Chemical
30	T049	Cell or Molecular Dysfunction	84	T001	Organism
31	T103	Chemical	85	T032	Organism Attribute

No.	TUI	STY	No.	TUI	STY
32	T120	Chemical Viewed Functionally	86	T040	Organism Function
33	T104	Chemical Viewed Structurally	87	T115	Organophosphorus Compound
34	T201	Clinical Attribute	88	T042	Organ or Tissue Function
35	T200	Clinical Drug	89	T046	Pathologic Function
36	T019	Congenital Abnormality	90	T101	Patient or Disabled Group
37	T060	Diagnostic Procedure	91	T121	Pharmacologic Substance
38	T047	Disease or Syndrome	92	T067	Phenomenon or Process
39	T203	Drug Delivery Device	93	T039	Physiologic Function
40	T111	Eicosanoid	94	T002	Plant
41	T018	Embryonic Structure	95	T098	Population Group
42	T069	Environmental Effect of Humans	96	T097	Professional or Occupational Group
43	T126	Enzyme	97	T192	Receptor
44	T050	Experimental Model of Disease	98	T014	Reptile
45	T099	Family Group	99	T006	Rickettsia or Chlamydia
46	T013	Fish	100	T184	Sign or Symptom
47	T168	Food	101	T054	Social Behavior
48	T021	Fully Formed Anatomical Structure	102	T110	Steroid
49	T169	Functional Concept	103	T061	Therapeutic or Preventive Procedure
50	T004	Fungus	104	T024	Tissue
51	T028	Gene or Genome	105	T010	Vertebrate
52	T045	Genetic Function	106	T005	Virus
53	T083	Geographic Area	107	T127	Vitamin
54	T131	Hazardous or Poisonous Substance			

Consistency of prioritization

We studied the consistency of using different types of biomedical records. For example, the number of causal genes ranked at the top was 1398 for OMIM and 1196 for PubMed using STY as the phenotype vocabulary filter. The number of top-ranked causal genes common to both was 1017, which represents a minimum overlap of 73% (1017/1398). The proportions of consistent prioritization results observed between any two phenotype sources with respect to different vocabulary controls are shown in Table S8, and those observed between any two vocabulary filters with respect to different phenotypic sources are shown in Table S9.

Table S8 Proportion of common testing sets highly ranked between phenotype sources.

	STY	MeSH	ICD9CM	GO
OMIM_PubMed	0.73	0.66	0.65	0.52
OMIM_GeneRIF	0.61	0.65	0.46	0.39
PubMed_GeneRIF	0.77	0.82	0.57	0.59

Table S9 Proportion of common testing sets highly ranked between vocabulary filters.

	OMIM	PubMed	GeneRIF
STY_MeSH	0.91	0.84	0.81
STY_ICD9CM	0.57	0.49	0.38
STY_GO	0.50	0.37	0.29
MeSH_ICD9CM	0.59	0.51	0.38
MeSH_GO	0.50	0.37	0.30
ICD9CM_GO	0.53	0.33	0.28

Finally, we studied the consistency of complex-based prediction with different confidence score thresholds to define the protein complexes. We show the common top-ranking testing sets between each pair of score thresholds in Figure S4. We observed that the common fraction between a pair of approximate thresholds was high. For instance, between confidence score 900 and confidence score 950, or between 500 and 600. However, two largely discrepant thresholds led to less agreement on accurate predictions, which might be because of a remarkable increase/decrease in the number of biomedical records linked to the altered size of the protein complexes.

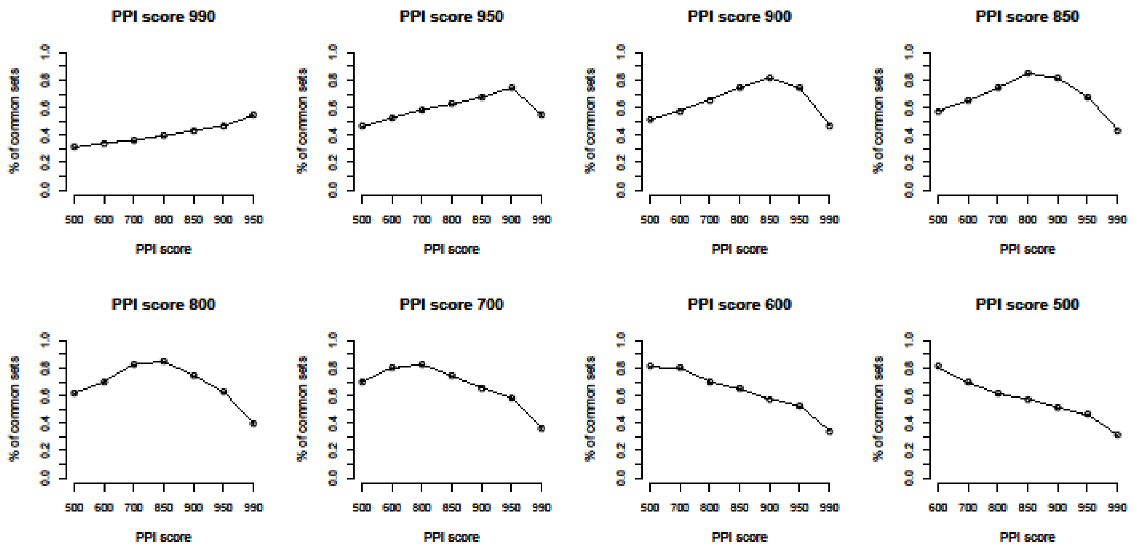

Figure S4 Consistency of prioritization for different confidence score thresholds.

Proportion of top-ranked causal genes

The proportion of testing sets for which the causal genes were ranked at the top or within the top five is shown in Table S10 for different combinations of phenotypes and vocabulary filters.

Table S10 Proportion of testing sets where the causal genes were highly ranked.

	OMIM		PubMed		GeneRIF	
	Top1	Top5	Top1	Top5	Top1	Top5
STY	0.52	0.81	0.44	0.74	0.38	0.65
MeSH	0.50	0.79	0.40	0.70	0.40	0.68
ICD9CM	0.40	0.74	0.34	0.62	0.33	0.65
GO	0.35	0.66	0.24	0.51	0.24	0.53

Differences in phenotypic profile

The number of associations between human genes and biomedical records are distinguishable for different types of biomedical records (Table S11). Each phenotypic document (e.g., a PubMed abstract) was converted into a term vector that was further standardized with a vocabulary filter (e.g., MeSH) before the semantic similarity with the

disease document (i.e., an OMIM record) was calculated. The average number of concepts/terms generated by a phenotype differed considerably (Table S12), and correlated (0.75) with prioritization ability (AUC). With a large volume of terms, matching words were found more easily using STY or MeSH than using ICD9CM or GO (Table S13).

Table S11 Overview of phenotype information for different phenotype sources.

	**No. of links between gene and phenotype**	**No. of Entrez genes**
**OMIM**	17933	14388
**PubMed**	473102	28207
**GeneRIF**	178009	11686

Table S12 Average number of concepts/terms per phenotype description after vocabulary filtering.

	STY	MSH	GO	ICD9CM
OMIM	147.5	94.2	10.8	8.2
PubMed	56.7	36.5	5.8	3.1
GeneRIF	6.2	3.9	1.5	1.1

Table S13 Volume of different vocabulary terms in the Unified Medical Language System.

	**STY**	**MSH**	**ICD9CM**	**GO**
**No. of concepts**	1426437	295842	48897	20306
